# Dissolution Rate and Stability Improvement of Hydrochlorothiazide and Valsartan by Co‐Amorphous Formulation

**DOI:** 10.1155/adpp/9387077

**Published:** 2026-09-30

**Authors:** Asal M. Abdulraheem, Khouloud A. Alkhamis, Suhair S. Al-Nimry

**Affiliations:** ^1^ Department of Pharmaceutical Technology Faculty of Pharmacy Jordan University of Science and Technology Irbid Jordan just.edu.jo

**Keywords:** co-amorphous, hydrochlorothiazide, powder dissolution, valsartan

## Abstract

The current study aimed to improve the dissolution rate and physical stability of two antihypertensive drugs, hydrochlorothiazide, and valsartan, through the development of a co‐amorphous system. A co‐amorphous system was successfully prepared at molar ratios of 1:1, 1:2, and 1:6 employing rotary evaporation technique. Characterization of the formulations was performed using powder X‐ray diffraction, differential scanning calorimetry, Fourier transform infrared spectroscopy, and thermogravimetric analysis. Powder X‐ray diffraction confirmed the amorphous nature of all samples, which remained physically stable under ambient dry conditions for at least 8 months. Differential scanning calorimetry confirmed the formation of a single‐phase system. The experimental glass transition temperature values revealed a negative deviation from those calculated by the Gordon–Taylor equation, indicating nonideal mixing and intermolecular interactions. Fourier transform infrared spectroscopy spectra supported these findings, showing peak broadening in regions corresponding to the functional groups of the parent drugs. The thermogravimetric analysis demonstrated that the samples were thermally stable, with no degradation observed up to 100°C. Dissolution studies performed in phosphate buffer (pH 6.8, 25°C) revealed significant enhancements in the dissolution rates of both hydrochlorothiazide and valsartan from the co‐amorphous formulations compared to their unprocessed forms and corresponding physical mixtures. A validated high‐performance liquid chromatography method was developed for the simultaneous analysis of hydrochlorothiazide and valsartan using a Shimadzu LC‐2030C 3D Plus system with a photodiode array detector. Separation was achieved on a Waters C18 column (250 × 4.6 mm, 5 μm) using a mobile phase of acetonitrile, methanol, and ammonium acetate buffer (20:50:30, v/v) at a flow rate of 0.70 mL/min. Detection wavelengths were set at 271 nm for hydrochlorothiazide and 249 nm for valsartan. The method showed good linearity in the range from 0.0033 to 0.0670 mM (3.3–67.0 μM), precision (RSD < 2%), and reproducibility, with hydrochlorothiazide and valsartan eluting at 3.5 and 7.2 min, respectively. At the first minute, hydrochlorothiazide release increased by approximately 8.5‐, 8.7‐, and 7.1‐fold from the 1:1, 1:2, and 1:6 co‐amorphous systems relative to the unprocessed drug, and by 4.5‐, 4.8‐, and 2.4‐fold, respectively, compared to the corresponding physical mixtures. For valsartan, the co‐amorphous systems showed 2.9‐, 2.6‐, and 1.9‐fold improvements over the unprocessed drug, and 1.5‐, 1.8‐, and 1.5‐fold improvements over the matching physical mixtures at the same molar ratios. Calcium carbonate was physically mixed with all samples to minimize gelation upon aqueous exposure. These results suggest that co‐amorphous formulation is a promising strategy for enhancing the dissolution rate and physical stability of hydrochlorothiazide and valsartan.

## 1. Introduction

The increasing availability of novel active pharmaceutical ingredients (APIs) has led to significant therapeutic advances across a broad spectrum of diseases. However, the physicochemical properties of many new drug candidates present substantial challenges in formulation and development. According to the Biopharmaceutics Classification System (BCS), Class II drugs are characterized by low aqueous solubility and high membrane permeability. The limited solubility of these drugs often results in poor oral bioavailability, thereby posing considerable obstacles for pharmaceutical scientists during formulation design [1]. Notably, it has been estimated that approximately 40% of newly synthesized chemical entities are lipophilic in nature, which further restricts their therapeutic efficacy due to their inadequate solubility in aqueous environments [2].

The amorphous form offers enhanced apparent solubility. However, this solubility advantage is counterbalanced by inherent limitations in physical stability. Compared to the crystalline state, the amorphous form exhibits increased internal energy, specific volume, and molecular mobility, all of which contribute to a greater tendency toward recrystallization under ambient or accelerated storage conditions [3, 4]. Amorphization is typically achieved by disrupting the crystal lattice of the API through solution‐based techniques, thermal processes, or mechanical activation [5]. The improved solubility of amorphous drugs arises from their disordered molecular arrangement, increased free volume, and reduced molecular packing efficiency, which facilitate water ingress and promote faster dissolution. Additionally, the elevated Gibbs free energy of the amorphous form drives rapid dissolution as the system seeks to reach a more thermodynamically stable state [6]. Nevertheless, despite their solubility advantages, amorphous formulations are often compromised by poor physical stability. Their high free volume and hygroscopic nature enhance their propensity for moisture uptake, while their thermodynamic instability leads to a risk of recrystallization during processing and storage, posing significant challenges for formulation development and commercial manufacturing [5].

Amorphous solid dispersion (ASD) is a concept first introduced in the early 1960s [7]. It involves the dispersion of one or more APIs within an inert carrier with a high glass transition temperature, such as polymers [8, 9]. The formed intermolecular interaction hinders the recrystallization tendency of amorphous drugs [8, 10]. However, the use of these polymers also has some drawbacks. They are hygroscopic, which lowers the glass transition of the binary mixture and eventually leads to phase separation and recrystallization [3]. Additionally, the amount of drug that can be loaded is limited, making it suitable only for low‐dose loading of potent drugs. Otherwise, toxicity problems may arise [11].

In contrast to traditional ASDs, the co‐amorphous (CAM) system represents a more recent advancement in formulation science, wherein two or more low‐molecular‐weight compounds are combined to form a homogeneous, single‐phase amorphous matrix. These systems are typically characterized by a single glass transition temperature (Tg) and the presence of specific intermolecular interactions which contribute to the physical stabilization of the amorphous form. CAMs can be formulated as drug–drug or drug–excipient combinations, offering enhanced physical stability and improved dissolution performance relative to conventional polymer‐based ASDs [11, 12]. Co‐amorphization offers an effective approach to stabilize amorphous drugs by forming a uniform glassy matrix with a low‐molecular‐weight co‐former. Unlike polymer‐based systems, it reduces excipient content and relies on specific intermolecular interactions to inhibit recrystallization. This strategy also enhances dissolution and bioavailability beyond that of both crystalline and conventional amorphous forms [13].

A major advantage of CAMs is their ability to achieve higher drug loading than polymer‐based ASDs. In ASDs, polymers typically accommodate no more than 30% drug content, which can compromise physical stability [14]. CAMs, by contrast, can incorporate drug loadings exceeding 50% while retaining amorphous stability throughout storage [15].

The co‐former could be an API, amino acids [16], bile acids [17], organic acids [18], urea [19], or sulfonic acids [20]. The CAM of drug–drug combination emerged for the first time with the synchronized release of two drugs is the naproxen–cimetidine CAM, in 2009 [6]. The successfully formed CAM of drug–drug has been reported in the literature; indomethacin–cimetidine [21], indomethacin–ranitidine [22], indomethacin–naproxen [23], naproxen–cimetidine [6], famotidine–ibuprofen [24], ritonavir–indomethacin [25], atenolol–hydrochlorothiazide (HTC) [26], atorvastatin with carvedilol and glibenclamide [27], atorvastatin–glipizide [28], nateglinide–metformin hydrochloride [29], irbesartan–atenolol [30], telmisartan and HTC [31], valsartan (VAL)–nifedipine [32], VAL–cilnidipine [33], simvastatin–ezetimibe [34], nifedipine–nimodipine [35], and nifedipine–paracetamol [36].

The purpose of this investigation is to improve the physical stability and dissolution rates of two drugs, namely, HTC and VAL, through the co‐amorphization technique. HTC and VAL are both BCS Class II drugs.

Although HTC and VAL are widely coprescribed for the management of hypertension, their poor aqueous solubility significantly limits their oral bioavailability. Current commercial formulations are primarily based on physical mixtures (PMs) and often rely on multiple excipients to enhance performance. In particular, gelation phenomena during dissolution represent a known challenge, typically addressed through the incorporation of complex excipient systems.

In this study, we introduce a novel CAM drug–drug formulation of HTC and VAL as an alternative strategy to conventional approaches. Unlike polymer‐based amorphous dispersions or nanosizing techniques, this work employs a polymer‐free CAM system based on specific molar ratios of the two APIs. The CAM of 1:1, 1:2, and 1:6 demonstrated significant improvements in both dissolution rate and physical stability.

A key innovation of this work lies in the systematic investigation of additive effects on gelation behavior, where excipients were evaluated individually to understand their impact on the dissolution process. This approach led to the identification of calcium carbonate (CaCO_3_) as an effective single functional additive capable of preventing gelation and enabling uniform drug release. This contrasts with conventional formulations that require multiple excipients to achieve similar performance.

Furthermore, the CAM demonstrated the ability to enhance dissolution of both drugs without reliance on complex formulation strategies, highlighting that the improved dissolution is attributed to the amorphous single‐phase nature of the system, in which HTC and VAL are interconnected through intermolecular interactions that inhibit recrystallization. This simplified and minimal‐excipient approach may provide a practical and scalable platform, with the potential to reduce the required dose and overall formulation burden.

Overall, this work provides a new perspective on addressing dissolution and gelation challenges in combination therapy through rational CAM design.

The selection of drug combinations for CAM is often guided by clinical or pharmacological relevance. The pairing of HTC and VAL exemplifies this rationale, offering enhanced efficacy in the treatment of moderate‐to‐severe hypertension. VAL exerts its antihypertensive effect by selectively blocking the Angiotensin II Type 1 receptor, thereby reducing vasoconstriction and aldosterone secretion. HTC complements this mechanism by promoting natriuresis through inhibition of sodium reabsorption in the distal tubules. The resulting pharmacodynamic synergy enhances blood pressure control and supports the clinical value of this combination [37].

The selection of the investigated molar ratios was performed through a stepwise formulation strategy considering the physicochemical characteristics of both drugs. HTC exhibits a strong tendency toward recrystallization after amorphization, which represents a major challenge in maintaining the amorphous state. Glass‐forming ability (GFA) is an important parameter governing the resistance of amorphous materials to crystallization, with compounds exhibiting low GFA showing a greater tendency to recrystallize. Therefore, the incorporation of a suitable co‐former capable of reducing molecular mobility and enhancing amorphous‐phase stability is essential [38].

HTC is a thiazide diuretic commonly used in combination therapies for hypertension. Its formulation is limited by poor aqueous solubility (0.70 mg/mL) [39], which impacts dissolution and bioavailability. HTC has a molecular weight of 297.74 g/mol and a glass transition temperature of 117.85°C–120°C [40]. It is typically classified as a BCS Class II drug, although some reports suggest Class IV due to low permeability [41].

VAL is an orally active API with a molecular weight of 435.519 g/mol and a very low water solubility of 0.0234 mg/mL. It faces formulation challenges due to its negligible aqueous solubility [42].

In this system, VAL contributes beyond its therapeutic effect by acting as an antiplasticizer. This stabilizing effect inhibits recrystallization, making VAL both an API and a functional stabilizer within the CAM matrix [33]. The role of VAL will be as a co‐former with a high glass transition temperature (76°C) [43]. In a previous study, the CAM of VAL and cilnidipine demonstrated good stability. The positive outcomes observed from using VAL as a co‐former led to its selection in additional studies [33].

Designing a CAM comprising HTC and VAL is expected to offer dual benefits: enhanced dissolution rate and improved physical stability.

## 2. Materials and Methods

### 2.1. Materials

HTC and VAL raw materials were kindly provided as gift samples by the DAR Al DAWA Pharmaceutical Company (Amman, Jordan). The HPLC mobile phase consisted of acetonitrile (HPLC grade, 99.9%, Fisher Chemical, USA), methanol (HPLC grade, 99.9%, Fisher Chemical, USA), and ammonium acetate (Analytical Reagent 98% assay, GCC, UK). All were purchased from a local chemical supplier located in Amman, Jordan.

In the rotary evaporation method, acetone (HPLC grade, 99.8%, Fisher Chemical, USA) was used as a solvent.

The gelation issue in the prepared CAMs occurred upon contact with water. To correct this issue, various excipients with both hydrophilic and hydrophobic characteristics were tested (Table 1). Calcium carbonate (CaCO_3_, analytical reagent grade; Xilong Scientific Co., Ltd., Guangdong, China) was used in the formulation to prevent gelation during dissolution studies. Potassium dihydrogen phosphate was obtained from ISOLAB, Germany.

**TABLE 1:** Classification of excipients by aqueous solubility characteristics.

Hydrophilic excipients	Hydrophobic excipients	Amphiphilic excipients
Asparagine	Magnesium aluminum silicate	Na‐Taurocholate
L‐Aspartic acid	Saccharin	Na‐dodecyl sulfate (SDS)
Glycerol	Span	Capryol 90
Na‐dichromate	Cinnamic acid	Tween
Succinic acid	Magnesium stearate	
Sodium chloride (NaCl)	CaCO_3_	
Calcium dihydrogen phosphate	Lauric acid	
Aerosil (colloidal SiO_2_)	L‐cysteine	
Magnesium Tri‐silicate		
Vanillin		
Calcium silicate		

### 2.2. Methods

#### 2.2.1. High‐Performance Liquid Chromatography

The detection and quantification of HTC and VAL were performed using a Shimadzu HPLC system (LC‐2030C 3D Plus, Japan) equipped with a photodiode array (PDA) detector. The maximum absorbance wavelengths (λmax) were determined to be 271 nm for HTC and 249 nm for VAL. Chromatographic separation was achieved using a Waters C18 column (250 × 4.6 mm, 5 μm particle size, USA), with a ternary mobile phase consisting of acetonitrile, methanol, and ammonium acetate buffer in a volumetric ratio of 20:50:30 (v/v). The analysis was conducted at a flow rate of 0.70 mL/min with an injection volume of 5 μL. The column oven temperature was maintained at 40°C, while the rack temperature was set at 5°C.

##### 2.2.1.1. Preparation of Standard and Binary Mixture Solutions

A combined standard stock solution of HTC and VAL was prepared at a molar ratio of 1:1 by accurately weighing 0.5 g of HTC and 0.7 g of VAL and dissolving them in distilled water to a final volume of 100 mL, yielding a binary mixture stock solution.

Working standard solutions were prepared by appropriate dilution of the stock solution with the mobile phase to obtain ten concentration levels within the linearity range (0.0033–0.0670 mM) for both HTC and VAL.

The resulting solutions contained both drugs at defined concentrations and were used for calibration and validation studies. All solutions were filtered through a 0.45‐μm membrane filter prior to HPLC analysis.

#### 2.2.2. Preparation of CAM System by Rotary Evaporation Method

For the preparation of the CAM HTC–VAL mixtures, PMs were prepared in 1:1, 1:2, and 1:6 molar ratios, then dissolved in acetone. Additional trials were also conducted at HTC–VAL molar ratios of 2:1 using the same method.

CAM HTC–VAL systems were prepared at molar ratios of 1:1, 1:2, 1:6, and 2:1. For the 1:1 ratio, 0.5 g of HTC and 0.73 g of VAL were dissolved in 10 mL of acetone. For the 1:2 ratio, 0.5 g of HTC and 1.46 g of VAL were dissolved in 10 mL of acetone. For the 1:6 ratio, 0.5 g of HTC and 4.38 g of VAL were dissolved in 10 mL of acetone. For the 2:1 ratio, 0.5 g of HTC and 0.37 g of VAL were dissolved in 10 mL of acetone. All quantities were calculated based on the required molar ratios using molecular weights of 297.73 g/mol for HTC and 435.528 g/mol for VAL.

The prepared solutions were subjected to solvent removal by a BUCHI Labortechnik AG rotary evaporator (9230 Flawil/Switzerland, Type F‐100 chiller, R‐100 rotary evaporator, I‐100 interface), according to the following conditions: a bath temperature of 45°C (B‐100 Bath), 100 mbar vacuum (V‐100), for 10 min. The obtained solid residues were collected and stored in sealed containers for further characterization following drying under vacuum (Precision Vacuum Oven Model 19, USA) at 25°C and 50 μm Hg for overnight.

All obtained samples were dried in a vacuum oven under the aforementioned conditions for two nights to ensure the complete removal of residual solvent prior to the characterization and dissolution studies.

#### 2.2.3. Calculating the Theoretical Glass Transition Temperature According to the Gordon–Taylor Equation

According to the Gordon–Taylor equation, the theoretical Tg of the CAM was calculated as follows [44]:

(1) TgCAM= WHTC TgHTC+K WVAL TgVAL WHTC+K WVAL,

where W_HTC_ and W_VAL_ represent the weight fractions of the components. K is a constant that depends on the components’ densities. The density values used for the calculation of the Gordon–Taylor constant (K) were the experimentally determined true densities of the amorphous forms of HTC and VAL, as the Gordon–Taylor model describes the glass transition behavior of an amorphous binary mixture.

The Gordon–Taylor constant (K) was calculated by the following equation:

(2) K=ρHTC·Tg,HTC   ρVAL·Tg,VAL,

Tg_CAM_ is the Tg (in Kelvin) of the CAM; Tg _HTC_ and Tg _VAL_ are Tgs (in Kelvin) of the single‐amorphous HTC and VAL, respectively [23].

#### 2.2.4. Powder X‐Ray Diffraction (PXRD)

PXRD patterns were recorded for the pure VAL, pure HTC, and the CAMs prepared in 1:1, 1:2, and 1:6 molar ratios. The analysis was carried out using a Rigaku Ultima IV X‐ray diffractometer (ID2528N, Japan) operating at 40 kV (200 VAC), with a Cu Kα radiation source. Data acquisition was performed in the range of 5°–60° 2θ, using a step size of 0.0200° 2θ and a scan speed of 4° 2θ/min.

#### 2.2.5. Differential Scanning Calorimetry (DSC) and Thermogravimetric Analysis (TGA)

Conventional DSC was employed to evaluate the thermal behavior of the pure drugs (HTC and VAL), as well as the CAMs prepared in 1:1, 1:2, and 1:6 molar ratios. The experiments were conducted using a DSC instrument (NETZSCH‐PROTEUS‐DSC 204F1, Germany). For each measurement, approximately 5 mg of the sample was accurately weighed and placed into a standard aluminum DSC pan, which was sealed with a pin‐holed lid to allow for vapor release while maintaining thermal contact.

The samples were heated from room temperature to 100°C at a heating rate of 10°C/min. This approach was designed to detect subtle glass transitions.

TGA was conducted to evaluate the thermal stability and weight loss behavior of the prepared CAMs at 1:1, 1:2, and 1:6 molar ratios. The experiments were performed using a TGA instrument (NETZSCH PROTEUS 209F1 Iris‐TGA instrument, Germany). Approximately 5 mg of each sample was placed in a platinum crucible and heated from room temperature to 100°C at a linear heating rate of 10°C/min under a nitrogen atmosphere.

#### 2.2.6. Fourier Transform Infrared Spectroscopy (FTIR)

The FTIR spectra were recorded over a wavenumber range of 4000 to 500 cm^−1^, using a total of 12 scans at a resolution of 4 cm^−1^. The analysis was performed using a SHIMADZU IRAffinity1 spectrometer (604–8511, Japan). Prior to measurement, each sample was blended with spectroscopic‐grade potassium bromide (Sigma, USA).

#### 2.2.7. Powder Dissolution Rate

Powder dissolution experiments were carried out after sieving the samples to obtain a controlled particle size range (0.180 mm > Ps > 0.063 mm). Each experiment was conducted in triplicate using United States Pharmacopeia (USP) Dissolution Apparatus II (paddle method).

The tested formulations included the unprocessed drugs (HTC and VAL), PMs at 1:1, 1:2, and 1:6 molar ratios, and CAMs prepared at the same molar ratios. To prevent gelation and enhance powder dispersion during dissolution, 90 mg of CaCO_3_ was physically mixed with each sample before testing. The dissolution medium consisted of 300 mL potassium dihydrogen phosphate (ISOLAB, Germany) buffer at pH 6.8, selected for its biorelevance to intestinal pH and established solubility of HTC and VAL, maintained at room temperature (25°C ± 2°C), and stirred at 90 RPM using a paddle apparatus under sink conditions. During the powder dissolution experiments, 5‐mL aliquots were withdrawn every two minutes over a total dissolution period of 15 min (5‐mL aliquots withdrawn at 1, 3, 5, 7, 9, 11, 13, and 15 min), without replacement of the withdrawn medium. Each withdrawn sample was immediately filtered using a PTFE syringe filter (0.45‐μm pore size). The filtrates were then either directly analyzed or appropriately diluted before quantification using the validated HPLC method.

Due to the rapid release of both drugs from the CAM formulations under these conditions, the standard 2‐h dissolution protocol was adjusted. Instead, a 15‐min test duration was implemented, with sampling intervals reduced to every 2 min rather than every 5 min. This modification was essential to capture early‐stage dissolution dynamics more precisely and to ensure reliable comparisons between formulations. The higher frequency of data collection within the first 15 min allowed for a more accurate characterization of the rapid release behavior induced by the combined effects of phosphate buffer and CaCO_3_.

#### 2.2.8. Sample Preparation

The final dosage form used in this study was a powdered formulation consisting solely of the APIs—HTC and VAL—along with calcium carbonate, which was incorporated specifically to address the gelation issue observed during dissolution testing. No other excipients, binders, or compression steps were involved in the formulation. The components were carefully weighed, then passed through a standard sieve (0.180 mm > Ps > 0.063 mm) to ensure uniform particle size distribution. The resulting powder blend was directly introduced into the dissolution medium without capsule filling or tablet compression, allowing evaluation of the powder dissolution behavior of the CAM system.

#### 2.2.9. Long‐Term Physical Stability Testing

Storage conditions were established in accordance with International Council for Harmonisation (ICH) Q1A (R2) guidelines for long‐term stability studies. Adequate quantities of CAMs (1:1, 1:2, and 1:6 molar ratios) and corresponding PMs were stored separately in tightly sealed containers containing silica gel desiccant to minimize moisture uptake, with storage maintained at 25°C ± 2°C and RH ≤ 10%. All containers were placed in an amber glass desiccator surrounded by fresh silica gel to provide additional protection from humidity and light. Physical stability was monitored by monthly PXRD analysis over an 8‐month period (Months 1–8).

## 3. Results and Discussion

### 3.1. High‐Performance Liquid Chromatography

Figure 1 presents the HPLC chromatogram illustrating the efficient separation of HTC and VAL. HTC eluted at 3.5 min with detection at 271 nm, while VAL eluted at 7.2 min and was detected at 249 nm, both showing well‐defined peaks.

**FIGURE 1:**
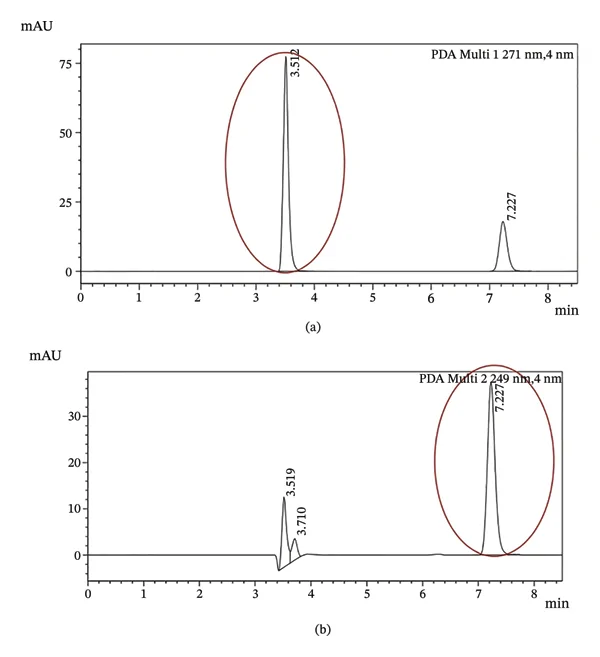
HPLC chromatogram showing the elution of (a) HTC at its λmax of 271 nm and (b) VAL at its λmax of 249 nm.

The analytical method employed in our project demonstrated accuracy, reliability, linearity, and reproducibility. Calibration curves were constructed by plotting concentration levels against the corresponding peak areas obtained at the *λ* max of each drug. Linearity was evaluated by calculating the intercept, slope, and correlation coefficient (*R* ^2^).

#### 3.1.1. Linearity

Linearity was assessed according to ICH Q2 (R1) guidelines across ten concentration levels ranging from 0.0033 to 0.0670 mM (3.3–67.0 μM) for both HTC and VAL. Calibration curves were constructed by plotting peak area versus concentration, and a strong linear relationship was observed for both drugs. As shown in Table 2, excellent linearity was obtained with correlation coefficients (*R* ^2^) ≥ 0.998 for both intraday and interday analyses. The slope and intercept values further confirm the reliability and consistency of the calibration model across the studied concentration range.

**TABLE 2:** Linearity and sensitivity parameters for HTC and VAL.

Analyte	Calibration type	*n*	Slope (m)	Intercept (b)	*R* ^2^	Linearity range (mM)	LOD (M)	LOQ (M)
HTC	Intraday	3	7 × 10^−6^	−323.76	0.999	0.0033–0.0670	5.896 × 10^−6^	1.787 × 10^−5^
HTC	Interday	3	8 × 10^−6^	−163.66	0.999	0.0033–0.0670	5.896 × 10^−6^	1.787 × 10^−5^
VAL	Intraday	3	5 × 10^−6^	−431.50	0.999	0.0033–0.0670	8.963 × 10^−6^	2.716 × 10^−5^
VAL	Interday	3	6 × 10^−6^	−175.12	0.998	0.0033–0.0670	8.963 × 10^−6^	2.716 × 10^−5^

These results meet the acceptance criteria recommended by ICH guidelines, supporting the suitability of the method for quantitative analysis. In addition, the limits of detection (LODs) and limits of quantification (LOQs) were determined. For HTC, the LOD and LOQ were 5.896 × 10^−6^ M and 1.787 × 10^−5^ M, respectively, while for VAL, the LOD and LOQ were 8.963 × 10^−6^ M and 2.716 × 10^−5^ M, respectively.

#### 3.1.2. Precision

The precision of the method was evaluated through intraday and interday analysis at ten concentration levels. As shown in Table 3, the %RSD values for both HTC and VAL were below or close to 2% in nearly all cases, indicating excellent repeatability and intermediate precision. These results are within the acceptable limits specified by Food and Drug Administration (FDA), ICH, and USP guidelines, which recommend %RSD values not exceeding 2% for analytical method precision. Minor deviations slightly above 2% were observed in isolated cases; however, these values remain acceptable and do not affect the overall precision of the method. This confirms the reliability and reproducibility of the proposed HPLC method [45–47].

**TABLE 3:** Precision results for HTC and VAL: intraday and interday %RSD (*n* = 3).

Concentration (*M* × 10^−3^)	HTC intraday RSD (%)	HTC interday RSD (%)	VAL intraday RSD (%)	VAL interday RSD (%)
0.0033	0.34	1.90	1.06	2.04
0.0083	0.42	0.89	0.39	1.46
0.0167	0.45	1.02	0.28	1.28
0.0251	0.37	1.03	0.32	1.38
0.0335	1.01	1.29	0.38	1.72
0.0368	0.39	2.02	0.37	1.56
0.0419	0.95	0.94	0.15	1.28
0.0502	0.36	0.84	0.44	1.90
0.0586	0.97	0.85	0.72	1.50
0.0670	0.26	1.25	0.27	1.91

#### 3.1.3. The Accuracy (Recovery)

The accuracy of the HPLC method was evaluated by analyzing spiked samples at three concentration levels corresponding to 80% (low), 100% (target), and 120% (high) of the nominal concentration for both HTC and VAL. As shown in Table 4, the recovery values for HTC ranged from 100.40% to 103.30%, while those for VAL ranged from 100.43% to 104.83%. These results fall within the acceptable limits specified by ICH Q2 (R1) guidelines, confirming the accuracy and suitability of the method for quantitative analysis.

**TABLE 4:** Accuracy results for HTC and VAL (recovery study).

Drug	Level	Concentration (M)	Recovery (%)
HTC	Low	2.514 × 10^−5^	103.30
HTC	Target	3.352 × 10^−5^	102.64
HTC	High	4.190 × 10^−5^	100.40
VAL	Low	2.514 × 10^−5^	104.83
VAL	Target	3.6872 × 10^−5^	100.43
VAL	High	5.028 × 10^−5^	104.59

#### 3.1.4. Robustness

The robustness of the HPLC method was evaluated by introducing small deliberate variations in analytical conditions, including changes in the pH of the mobile phase and the temperature of the sample tray. The effect of these variations on chromatographic performance was assessed in terms of peak characteristics and calibration parameters.

No significant changes were observed in retention time, peak shape, or calibration results under the modified conditions. These findings indicate that the method is robust and reliable, and minor variations in experimental parameters do not affect its performance [48, 49].

### 3.2. PXRD for the Pure Drugs, Successful, and Unsuccessful CAMs

The successful CAMs were prepared by the rotary evaporator at 1:1, 1:2, and 1:6 molar ratios. The 2:1 molar ratio did not yield 100% CAM. Instead, its PXRD pattern showed distinct Bragg peaks, confirming residual crystallinity and indicating failure to form a single‐phase CAM system. Therefore, this ratio was excluded from further analysis and was not included in subsequent characterization or dissolution studies. The PXRD diffractograms of the prepared CAMs are presented in Figure 2, while the diffractograms of the unprocessed pure drugs HTC and VAL are provided in Figure 3 as reference materials.

**FIGURE 2:**
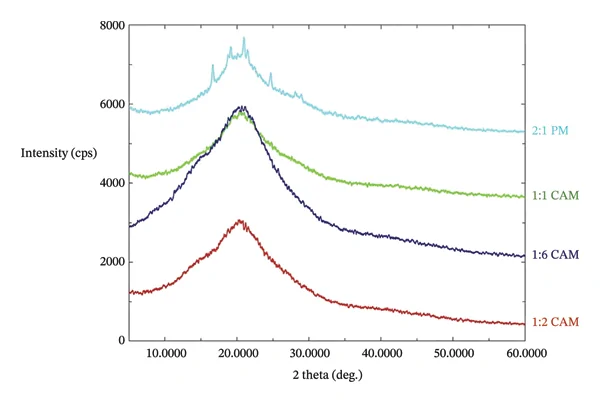
PXRD spectra of 1:1, 1:2, and 1:6 CAM and 2:1 PM molar ratio prepared by rotary evaporation.

**FIGURE 3:**
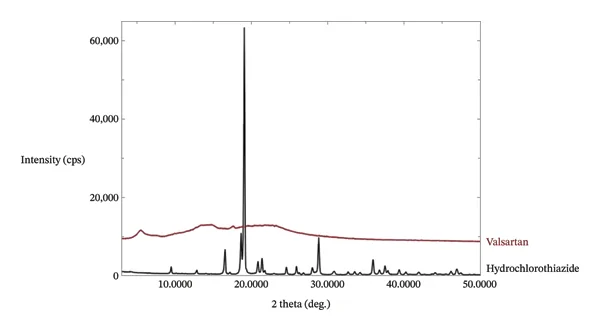
PXRD diffractograms of unprocessed hydrochlorothiazide and valsartan.

Accordingly, formulation development was initiated with an equimolar formulation (1:1 HTC:VAL), as equimolar compositions are widely used as the starting point in the design of binary CAM systems due to the possibility of forming equimolar heterodimers and promoting molecular‐level homogeneity [23]. After successful formation of the 1:1 CAM system, the proportion of VAL was increased to 1:2 and subsequently to 1:6 (HTC:VAL). This approach was based on the hypothesis that increasing the amount of VAL as a co‐former could provide additional molecular interactions and improve the stabilization of HTC by reducing molecular mobility and suppressing recrystallization [50]. Both formulations successfully produced CAM systems with improved physicochemical properties.

Importantly, the opposite compositional direction was also investigated by preparing an HTC‐rich formulation at a 2:1 (HTC:VAL) molar ratio. However, this formulation failed to generate a CAM system, as confirmed by PXRD, which indicated recrystallization of the components. This result supports the assumption that, due to the high recrystallization tendency of HTC, the lower concentration of VAL in this formulation was insufficient to provide adequate stabilization of the amorphous phase (Figure 2).

### 3.3. DSC and TGA for Prepared CAMs

In the CAM prepared at a 1:1 molar ratio, the Tg is observed as a minor step or inflection point. Analysis using NETZSCH Proteus software identified the inflection point for the 1:1 CAM at 53.0°C, with the glass transition onset Tg determined at 45.5°C. For 1:2 CAM, the analysis identified the inflection point at 50.7°C, with the onset Tg determined at 49.5°C. The 1:6 CAM exhibited a glass transition onset at 51.3°C with an inflection point at 64.2°C during the heating run. The DSC and TGA thermograms of the HTC–VAL CAM formulations are presented in Figure 4, including the 1:1 (A), 1:2 (B), and 1:6 (C) HTC:VAL molar ratios, while the DSC thermograms of the unprocessed pure drugs HTC and VAL are provided in Figure 5 as reference materials. Table 5 presents both the theoretical Tgs calculated using the Gordon–Taylor equation and the experimentally determined values. Table 5 shows that as the proportion of VAL content in the mixture increases, the theoretical Tg decreases. This trend is expected, as the Gordon–Taylor equation demonstrates that Tg depends on the weight ratio of each component. As the VAL content increases from 1:1 to 1:6 CAMs, the calculated Tg decreases, reflecting the lower Tg of VAL (76°C) compared to Tg of HTC (120°C) [33, 41].

FIGURE 4(A) DSC and TGA profiles of CAM 1:1 prepared by rotary evaporation at a heating rate of 10°C/min up to 100°C. (B) DSC and TGA profiles of CAM 1:2 prepared by rotary evaporation at a heating rate of 10°C/min up to 100°C. (C) DSC and TGA profiles of CAM 1:6 prepared by rotary evaporation at a heating rate of 10°C/min up to 100°C.

**Figure:**
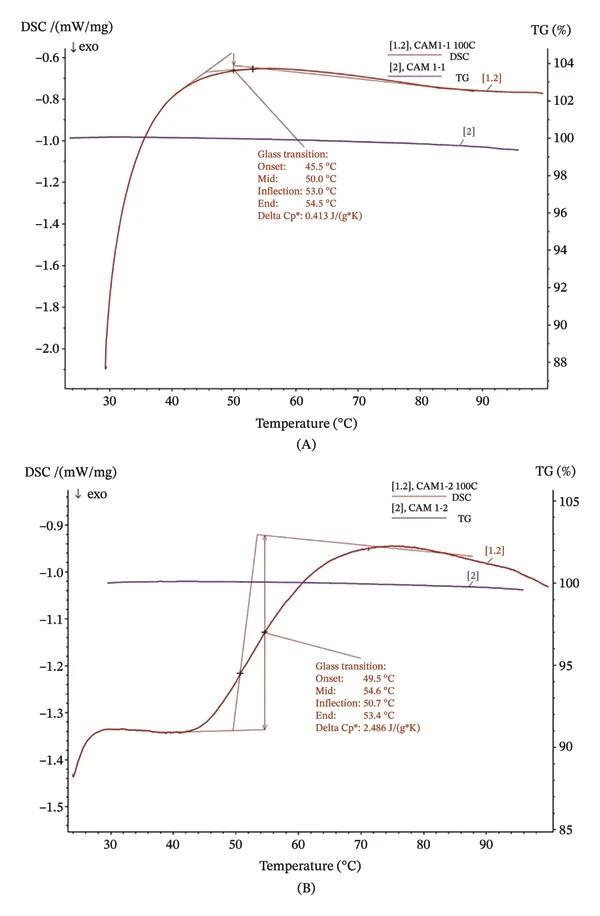

**Figure:**
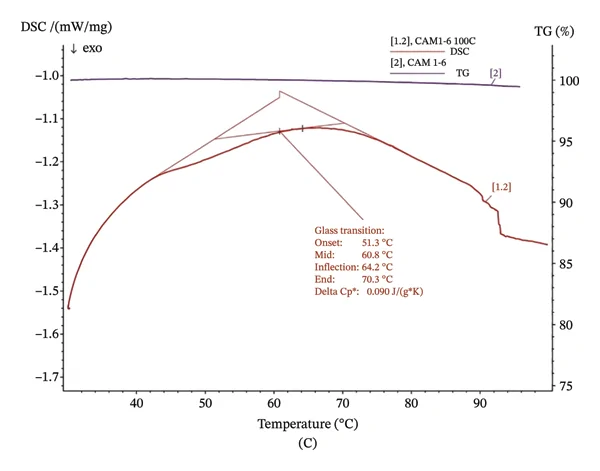

**FIGURE 5:**
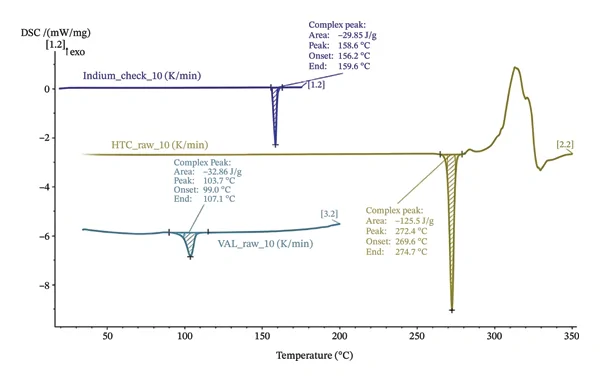
DSC thermograms of indium, unprocessed hydrochlorothiazide and valsartan.

**TABLE 5:** Negative deviation of experimental Tg from the theoretical Tg.

Molar ratio CAM (HTC: VAL)	Experimental Tg (°C)	Theoretical Tg (°C)	Negative deviation ΔTg (°C)
1:1	53	89.5	36.5
1:2	50.7	83.7	33.0
1:6	64.2	78.3	14.1

Table 6 presents the parameters used to calculate the theoretical glass transition temperature (Tg) using the Gordon–Taylor equation.

**TABLE 6:** Parameters used for the calculation of the theoretical glass transition temperature (Tg) of HTC–VAL co‐amorphous systems using the Gordon–Taylor equation.

CAM molar ratio (HTC:VAL)	Mass (g) HTC: VAL	Weight fraction HTC (*ω*_1_)	Weight fraction VAL (*ω*_2_)	Tg theoretical (K)	Tg theoretical (°C)	Tg experimental (°C)
1:1	0.5:0.7	0.4167	0.5833	∼362.6	∼89.5	53.0
1:2	0.5:1.4	0.2632	0.7368	∼356.8	∼83.7	50.7
1:6	0.5:4.2	0.1064	0.8936	∼351.5	∼78.3	64.2

The experimental Tg values exhibited a negative deviation from the theoretical Tg calculated using the Gordon–Taylor equation. This deviation arises from specific intermolecular interactions such as hydrogen bonding, which are not accounted for in the equation’s assumption of ideal miscibility and free volume additivity [44].

Considering the negative deviation between the experimental and theoretical Tgs, the extent of deviation decreases with increasing VAL content. In the 1:6 CAM formulation, the substantial excess of VAL, recognized for its antiplasticizing properties, appears to limit the depression of Tg. This effect is likely due to VAL’s ability to enhance the rigidity of the amorphous matrix, thereby reducing segmental mobility and contributing to improved structural stability.

TGA was conducted on CAM of HTC and VAL in three different molar ratios (1:1, 1:2, and 1:6), using a heating rate of 10 k/min from ambient temperature up to 100°C. This temperature range was selected to detect solvent evaporation or early‐stage decomposition, providing insight into the physical stability and residual solvent content of the CAMs. As illustrated in Figure 4A–C, the CAM formulations demonstrated thermal stability throughout the examined range, with no observable weight loss, indicating the absence of decomposition or volatilization processes below 100°C.

The experimentally determined true densities employed in the calculation of the theoretical Tg were as follows:

• Amorphous HTC density (ρHTC): 1.5058 g/mL
• Amorphous VAL density (ρVAL): 1.1351 g/mL

The calculated K value was as follows:= (1.5058 ^∗^ 393.15)/(1.1351 ^∗^ 348.15) ≈ 1.50.

### 3.4. FTIR for Prepared CAM and Unprocessed Drugs

The FTIR spectra of the individual pure drugs (HTC and VAL), their PM at a 1:1 molar ratio, and the CAMs prepared at 1:1, 1:2, and 1:6 molar ratios were recorded. The spectra of HTC, VAL, and PM are presented in Figures 6 and 7.

**FIGURE 6:**
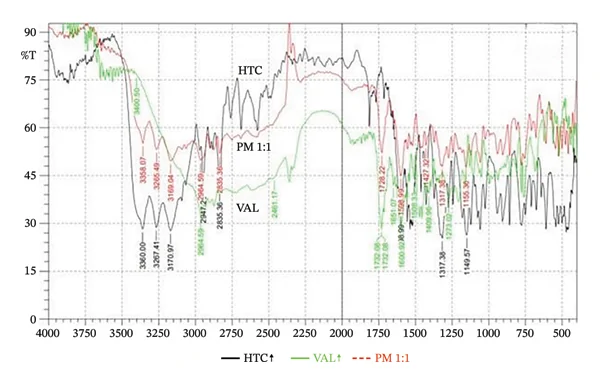
FTIR spectra of hydrochlorothiazide, valsartan, and PM 1:1.

**FIGURE 7:**
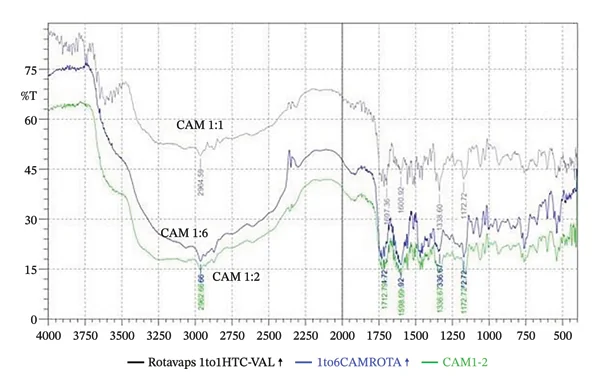
FTIR spectra of CAM 1:1, 1:2, and 1:6 prepared by rotary evaporation.

The FTIR spectra of the PM with HTC:VAL ratios of 1:1 exhibit an additive pattern, with no significant peak shifts in the characteristic functional groups (O–H/N–H, C=O, S=O), nor any substantial band broadening or disappearance. These observations indicate the absence of intermolecular interactions between the two drugs within the PM.

The FTIR spectrum of the 1:1, 1:2, and 1:6 CAM formulations, prepared via rotary evaporation and presented in Figure 7, reveals noticeable deviations from the spectra of the pure drugs. To facilitate interpretation, these spectral differences are discussed in detail across relevant wavenumber regions.

In the 3500–3000 cm^−1^ region, the FTIR spectrum of the CAM formulation exhibits a noticeable peak disappearance accompanied by band broadening and reduced peak resolution. These spectral changes indicate the presence of strong hydrogen bonding, likely involving the hydroxyl group of the carboxylic acid in VAL and the sulfonyl (S=O) and amino (–NH_2_) groups of HTC. The observed broadening further supports the existence of significant intermolecular interactions between the two components. In the 1700–1750 cm^−1^ region, the FTIR spectrum of the CAM formulation exhibits a reduction in intensity and a shift to lower wavenumbers of the carbonyl (C=O) stretching band associated with the carboxylic acid group in pure VAL. These spectral changes provide evidence for the involvement of the carbonyl group of the –COOH moiety in hydrogen bond formation with HTC. In the 1600–1650 cm^−1^ region, the FTIR spectrum of the CAM formulation displays significant weakening of the amide carbonyl (C=O) stretching band observed in pure VAL, with partial overlap from the aromatic C=C stretching vibrations present in both components. This spectral behavior suggests enhanced stabilization of the CAM, potentially mediated by hydrogen bonding interactions involving the amide carbonyl group of VAL. In the 1100–1300 cm^−1^ region, the FTIR spectrum of the CAM formulation reveals noticeable broadening and attenuation of the symmetric and asymmetric stretching vibrations of the sulfonamide S=O group. These spectral changes indicate the involvement of the sulfonyl functional group of HTC in hydrogen bond formation within the CAM. Collectively, the formation of hydrogen bonds in the 1:1, 1:2, and 1:6 CAM formulation contributes significantly to the stabilization of the CAM.

Since the VAL content in CAM 1:2 and 1:6 is higher, the interaction capacity of HTC appears to be exceeded, resulting in some functional groups of VAL remaining unbound with no corresponding interaction partner. This is reflected in the FTIR spectra by the reduced peak broadening as the molar ratio of VAL increases.

### 3.5. Powder Dissolution Rate

Upon exposure to aqueous media, the CAM samples exhibited strong hydration and rapid swelling, followed by self‐association into irregular shapes and coaggregation into viscous, gel‐like structures. These observations, as shown in Figure 8, are indicative of gelation behavior in the prepared CAMs, an issue that poses a significant challenge for efficient drug dissolution.

**FIGURE 8:**
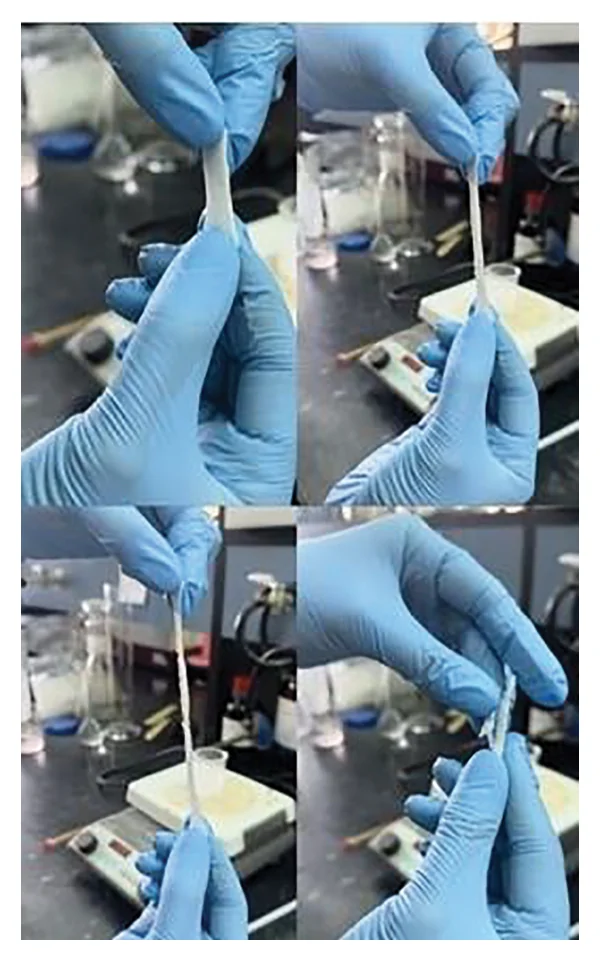
In‐laboratory observation of gelation phenomena in CAM 1:1.

Several mechanisms may explain how gelation interferes with drug release from the prepared CAMs:

1. The gel structure formed upon hydration can physically trap both drugs within the swollen mass, significantly hindering their release into the dissolution medium particularly in the CAM formulations, where drug–co‐former interactions are more pronounced [51].
2. Gelation leads to the development of a viscous, semisolid layer surrounding the particles. This layer acts as a diffusion barrier, reducing the available surface area for interaction with the dissolution medium and slowing down drug release.
3. The self‐associated structures formed during gelation tend to exhibit irregular shapes, resulting in inconsistent and variable surface areas available for dissolution [52].

Gelation in CAM has been rarely reported, but notable examples include studies in 2022 by Chen et al. [53], who observed gelation in CAM of sinomenine with platensimycin or sulfasalazine. In 2023, Jiawei Han et al. [54] reported similar behavior in CAM resveratrol–piperine systems, and in 2024, Feiyan Pu et al. [55] documented gelation in CAM taxifolin–oxymatrine formulations. Despite these observations, gel formation remains a relatively uncommon phenomenon and has only recently been reported in the literature by researchers studying CAMs.

The proposed mechanism behind gelation in CAM is the presence of strong intermolecular interactions between the drug and the co‐former. These interactions make the amorphous powder highly sensitive to water or moisture exposure, promoting the formation of an interconnected molecular network in three‐dimensional space. FTIR spectra of the CAMs support the existence of such interactions, as evidenced by characteristic shifts and changes in peak intensities. Furthermore, this behavior is visibly confirmed during contact with water, where the sample rapidly transforms into a viscous, gel‐like structure, consistent with gelation phenomena. Theoretically, gelation can be described as the formation of a three‐dimensional network structure that arises through multiple hierarchical levels of molecular assembly, progressing from individual molecules to a fully interconnected framework. In the present study, when the CAM was introduced into an aqueous environment, it initially floated and aggregated at the surface, gradually forming a soft, sticky mass [56].

Upon further hydration and partial dissolution, this mass exhibited tensile and elastic properties—when stretched or compressed manually, it showed noticeable toughness and flexibility, which are hallmark characteristics of gels. Such materials exhibit both the elasticity of solids and the viscosity of liquids [52, 57, 58].

Gelation in CAM was found to be temperature‐dependent, with gel formation becoming more pronounced at elevated temperatures. As the temperature increased, particularly toward physiological conditions (37°C), the extent of gelation also increased. This observation prompted the decision to conduct dissolution experiments at 25° [52].

From a preformulation standpoint, the objective was to evaluate the inherent dissolution behavior of the CAM without incorporating stabilizing excipients during the preparation of the CAM. Therefore, excipients were excluded from the rotary evaporation process and instead were physically mixed with the CAM prior to the dissolution experiments. This approach ensured that the excipient did not influence the preparation of the CAM but was instead used solely to assess its impact on dissolution performance under test conditions.

To address the gelation issue, several additives were evaluated for their ability to prevent or reduce gel formation. The only additive that successfully resolved the gelation problem at 25°C was CaCO_3_. CaCO_3_ likely prevents the initial adsorption of water onto the CAM surface, thereby disrupting the cascade of molecular events that leads to the formation of an interconnected gel matrix.

While previous studies have addressed the gelation issue by incorporating additives directly into the CAM components prior to the co‐amorphization process, the current project aims to resolve the problem without altering the CAM formulation itself. This finding is particularly noteworthy, as CaCO_3_ was not incorporated during the CAM preparation itself but was instead added in a small amount (not exceeding 90 mg) as PM.

Visual observations of the mixtures containing hydrophilic excipients formed larger aggregates, which readily adhered to the stirring paddles and absorbed water rapidly. In contrast, when hydrophobic excipients were used, powder aggregation was substantially reduced. It is hypothesized that hydrophobic particles adhered to the surface of the HTC–VAL CAMs, forming a barrier that limited interparticle contact and slowed water penetration, thereby suppressing gelation. This behavior suggests that the surface characteristics of excipients, particularly their wettability and interfacial compatibility with the amorphous drug, play a critical role in modulating gelation behavior.

Dissolution profiles of the CAMs of HTC and VAL separately were compared with their corresponding PMs and with unprocessed HTC and VAL using the regulatory‐adjusted similarity factor (*f*_2_) and unpaired *t*‐test. Adjusted *f*_2_ calculations were performed according to ICH/FDA recommendations, retaining only one time point after both formulations surpassed 85% dissolution, Tables 7 and 8.

**TABLE 7:** Adjusted *f*_2_ and unpaired *t*‐test results for HTC in CAM dissolution compared with unprocessed HTC and PM.

Molar ratio	Comparison	Adjusted similarity factor (*f*_2_)	Interpretation (*f*_2_)	*p*‐value (*t*‐test)	Statistical significance
1:1	CAM vs. HTC	18.3	Dissimilar	< 0.05	Significant
	CAM vs. PM	22.9	Dissimilar	< 0.05	Significant

1:2	CAM vs. HTC	13.2	Dissimilar	< 0.05	Significant
	CAM vs. PM	29.5	Dissimilar	< 0.05	Significant

1:6	CAM vs. HTC	20.4	Dissimilar	< 0.05	Significant
	CAM vs. PM	47.12	Dissimilar	< 0.05	Significant

**TABLE 8:** Adjusted *f*_2_ and unpaired *t*‐test results for VAL in CAM dissolution compared with unprocessed VAL and PM.

Molar ratio	Comparison	Adjusted similarity factor (*f*_2_)	Interpretation (*f*_2_)	*p*‐value (*t*‐test)	Statistical significance
1:1	CAM vs. VAL	46.3	Dissimilar	< 0.05	Significant
	CAM vs. PM	36.1	Dissimilar	< 0.05	Significant

1:2	CAM vs. VAL	42.7	Dissimilar	< 0.05	Significant
	CAM vs. PM	39.8	Dissimilar	< 0.05	Significant

1:6	CAM vs. VAL	40.2	Dissimilar	< 0.05	Significant
	CAM vs. PM	43.9	Dissimilar	< 0.05	Significant

#### 3.5.1. HTC Powder Dissolution in pH 6.8 Phosphate Buffer With Calcium Carbonate: 15‐Minute Release Profile

The powder dissolution results for HTC were analyzed across different formulations, including unprocessed crystalline HTC, PMs at 1:1, 1:2, and 1:6 molar ratios, and CAMs prepared at the same ratios. To facilitate a clearer understanding of the data, each CAM formulation was compared directly with its corresponding PM and with unprocessed HTC.

As shown in Figure 9, the release profile of HTC from the CAM 1:1 formulation was compared with that of the PM 1:1 and unprocessed crystalline HTC. The dissolution of HTC from unprocessed crystalline powder exhibited the lowest percentage of drug release across all time points, reaching only 24.6% within 15 min. This result confirms the inherently slow‐release rate of HTC in its crystalline form. A marked improvement in HTC release was observed from the CAM 1:1 formulation, with an initial release of 34.8% within the first minute, reaching 80.6% by the end of the 15 min. This substantial enhancement highlights the critical role of co‐amorphization in improving dissolution performance, particularly in the early stages of release.

**FIGURE 9:**
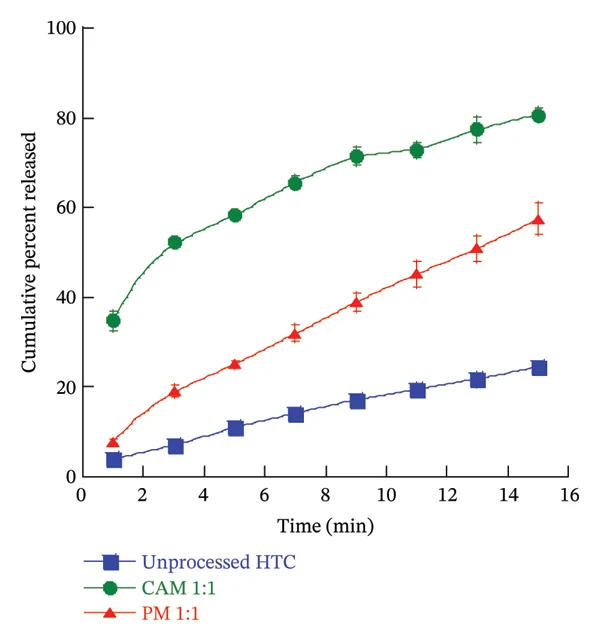
Percentage powder dissolution profile of unprocessed HTC, and from 1:1 PM, 1:1 CAM in phosphate buffer pH 6.8 with CaCO_3_, at 25°C and 90 rpm, *n* = 3.

In a 1:2 molar ratio, a consistent trend was observed; CAM 1:2 exhibited the highest dissolution, achieving 100% release within 15 min, followed by PM 1:2 with 80.7%, and the slowest release from unprocessed HTC (Figure 10).

**FIGURE 10:**
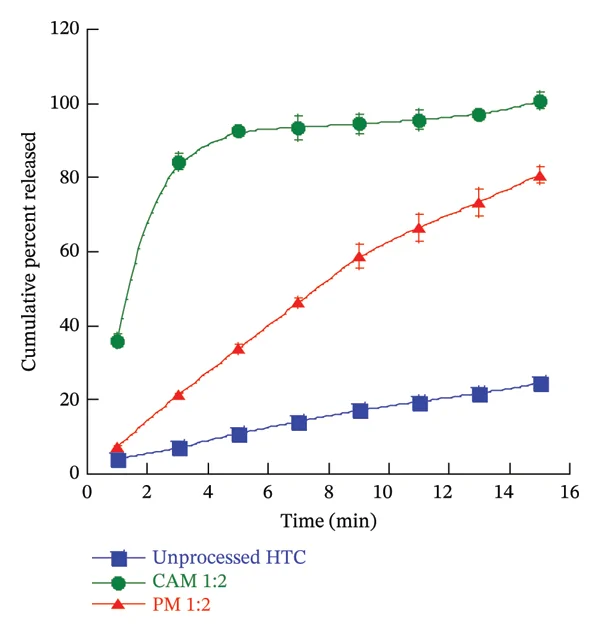
Percentage powder dissolution profile of unprocessed HTC, and from 1:2 PM, 1:2 CAM in phosphate buffer pH 6.8 with CaCO_3_, at 25°C and 90 rpm, *n* = 3.

A similar trend was observed in CAM 1:6, which exhibited the highest dissolution rate, achieving 100% release within 15 min. This was followed by PM 1:6, which reached 81.5%, while the lowest release was recorded for the unprocessed HTC (Figure 11).

**FIGURE 11:**
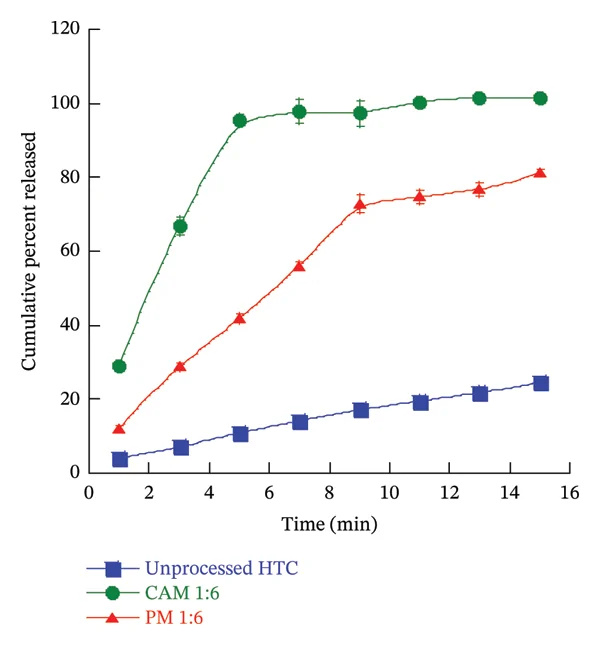
Percentage powder dissolution profile of unprocessed HTC, and from 1:6 PM, 1:6 CAM in phosphate buffer pH 6.8 with CaCO_3_, at 25°C and 90 rpm, *n* = 3.

The results clearly demonstrate a significant improvement in the dissolution of HTC from all CAM formulations when compared to unprocessed crystalline HTC. Specifically, CAM 1:2 and CAM 1:6 achieved complete (100%) drug release within 15 min, while CAM 1:1 reached 80.6%. In contrast, unprocessed HTC exhibited only 24.6% release within the same timeframe. These findings confirm the effectiveness of co‐amorphization in improving the dissolution performance of HTC. Furthermore, they demonstrate that CaCO_3_ successfully suppressed gelation.

#### 3.5.2. VAL Powder Dissolution in pH 6.8 Phosphate Buffer With Calcium Carbonate:15‐Minute Release Profile

The graph in Figure 12 demonstrates that VAL release from the CAM 1:1 formulation exhibits the fastest initial dissolution rate compared to both PM 1:1 and unprocessed amorphous VAL, achieving 74% release within the first 3 min, while PM 1:1 and unprocessed VAL reached 48.3% and 41.6%, respectively. This observation supports the effectiveness of the co‐amorphization technique in enhancing early‐stage dissolution of VAL. However, the CAM 1:1 system displayed a release plateau after 11 min, stabilizing at approximately 90%, whereas unprocessed VAL continued to release beyond this point, ultimately outperforming CAM 1:1 over the full dissolution period. This suggests that while co‐amorphization improves the rate of initial dissolution, it may introduce barriers to complete release—possibly due to matrix formation or localized viscosity changes.

**FIGURE 12:**
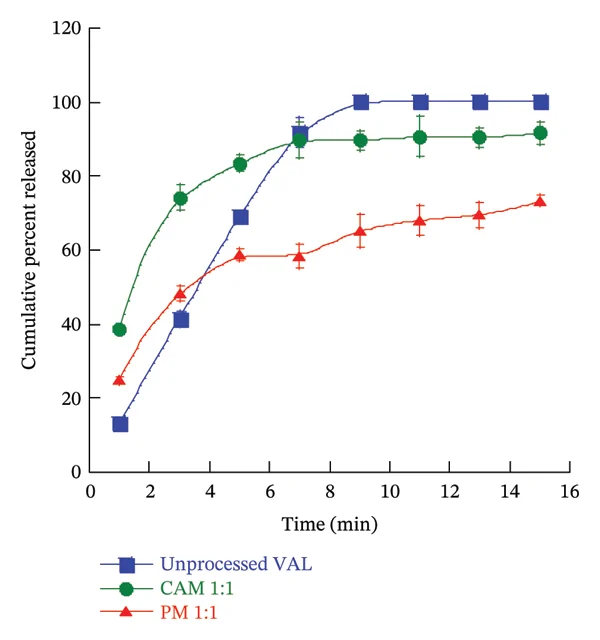
Percentage powder dissolution profile of unprocessed VAL, and from 1:1 PM, 1:1 CAM in phosphate buffer pH 6.8 with CaCO_3_, at 25°C and 90 rpm, *n* = 3.

The PM 1:1 formulation exhibited the lowest percentage release and the slowest dissolution rate for VAL, indicating that the presence of crystalline HTC may hinder the release of amorphous VAL when the two are simply blended in the PM. This result suggests that drug–drug interactions in the PM may negatively impact VAL dissolution, despite its amorphous state. These findings further underscore the importance of co‐amorphization as a promising formulation strategy, which enables a more uniform molecular dispersion and improved dissolution performance compared to conventional physical mixing.

The CAM 1:2 formulation exhibited the fastest release of VAL compared to both the unprocessed amorphous VAL and the PM 1:2 (Figure 13). It achieved 34.8% release at the onset of the experiment and reached complete release (100%) within 15 min. Throughout the entire dissolution period, CAM 1:2 consistently outperformed PM 1:2, which failed to achieve complete release. These results highlight the effectiveness of co‐amorphization at the 1:2 molar ratio in significantly enhancing both the rate and extent of VAL dissolution.

**FIGURE 13:**
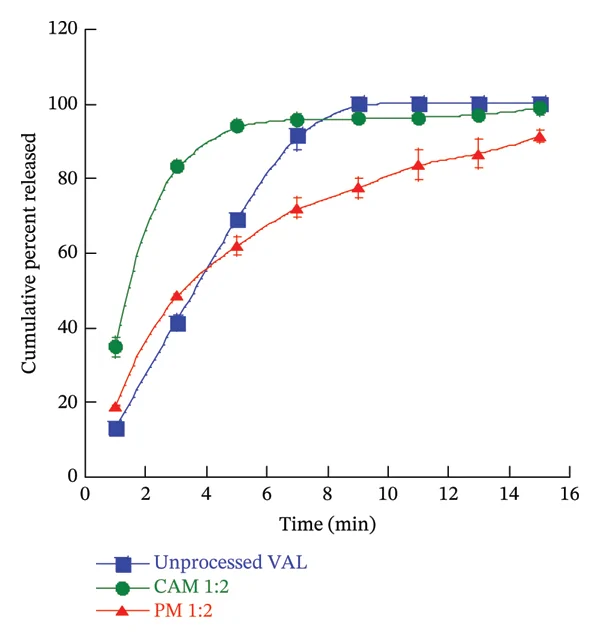
Percentage powder dissolution profile of unprocessed VAL, and from 1:2 PM, 1:2 CAM in phosphate buffer pH 6.8 with CaCO_3_, at 25°C and 90 rpm, *n* = 3.

The dissolution curves of PM 1:6 and unprocessed amorphous VAL were nearly identical across all time points, with both formulations reaching approximately 43% release within the first 3 minutes. In contrast, CAM 1:6 began with a 25% release in the first minute and achieved complete release within 7 min. These results clearly indicate that CAM 1:6 exhibited the fastest dissolution in the early stages, outperforming both the PM and unprocessed VAL in terms of release rate (Figure 14).

**FIGURE 14:**
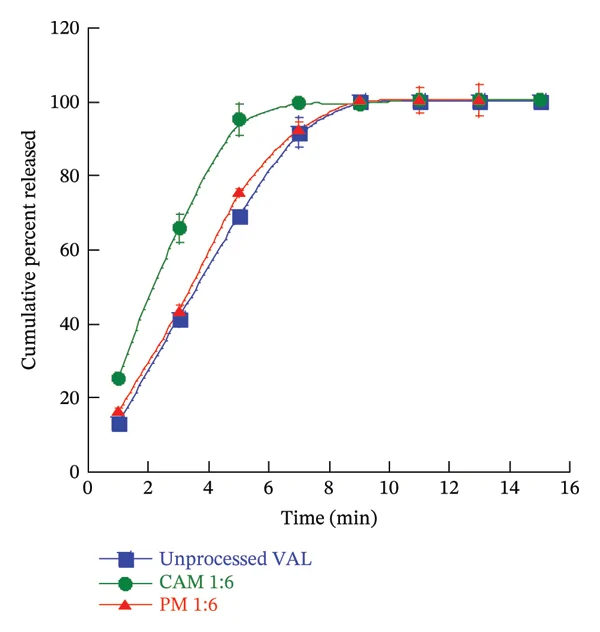
Percentage powder dissolution profile of unprocessed VAL, and from 1:6 PM, 1:6 CAM in phosphate buffer pH 6.8 with CaCO_3_, at 25°C and 90 rpm, *n* = 3.

The results clearly demonstrate a significant improvement in VAL dissolution from CAM formulations during the first 7 min when compared to the unprocessed amorphous VAL.

All in all, in the first minute, HTC release increased by approximately 8.5‐, 8.7‐, and 7.1‐fold from the 1:1, 1:2, and 1:6 CAM systems relative to the unprocessed drug, and by 4.5‐, 4.8‐, and 2.4‐fold, respectively, compared to the corresponding PMs. For VAL, the CAM systems showed 2.9‐, 2.6‐, and 1.9‐fold improvements over the unprocessed drug, and 1.5‐, 1.8‐, and 1.5‐fold improvements over the matching PMs at the same molar ratios.

### 3.6. Physical Stability Test

PXRD patterns were collected periodically to monitor any potential recrystallization or physical transformations during storage. Each CAM sample (1:1, 1:2, and 1:6 molar ratios) was analyzed using PXRD at one‐month, two‐month, three‐month, and 8‐month intervals. The diffractograms of all analyzed samples consistently exhibited a broad “halo” pattern, with no sharp crystalline peaks observed. The persistence of the halo pattern across all time points and compositions demonstrates that no recrystallization or phase transformation was observed under the tested storage conditions (dry place [RH ≤ 10%], 25°C). The results also confirm that the co‐amorphization technique effectively maintains both components in the amorphous state.

## 4. Conclusion

The objective was to prepare CAMs of HTC and VAL, both classified as BCS Class II drugs, to enhance their dissolution rate and physical stability in the amorphous state. This objective was successfully achieved through the preparation of a single‐phase CAM.

These systems were robustly engineered using the rotary evaporation method at modulating molar ratios (1:1, 1:2, and 1:6 of HTC: VAL). The obtained CAMs were comprehensively characterized utilizing PXRD and DSC, guaranteeing the formation of stable CAMs that preserved their physical integrity for at least 8 months under ambient dry storage conditions. Interestingly, the obtained Tgs of the CAMs from the DSC analysis reflected a negative deviation from the theoretical values predicted by the Gordon–Taylor equation, revealing the nonideal mixing and the formation of intermolecular interactions between the two drugs, HTC and VAL. The observed outcome was further reinforced by FTIR, which displayed peak broadening in the resulting spectra of the CAM compared to the individual drugs, which evidently supports the molecular‐level interactions.

The powder dissolution results showed that both HTC and VAL exhibited rapid and complete dissolution in the CAM mixtures prepared at 1:2 and 1:6 molar ratios.

The unexpected gelation observed upon contact with aqueous media at 25°C was successfully resolved through physical mixing of the CAM sample with hydrophobic CaCO_3_. This solution was identified after extensive testing of both hydrophilic and hydrophobic additives.

For future work, we recommend evaluation of additional hydrophobic excipients to prevent gelation at physiological temperature (37°C).

The present study was designed to investigate the preparation and physicochemical characterization of HTC–VAL CAM systems, including their solid‐state properties, physical stability, and dissolution behavior, whereas evaluation of the relationship between molar ratio and therapeutic performance through pharmacokinetic and pharmacodynamic studies will be the focus of future investigations.

## Funding

The authors would like to thank the Deanship of Scientific Research at Jordan University of Science and Technology for financial support (Grant No. 44/2024).

## Conflicts of Interest

The authors declare no conflicts of interest.

## Data Availability

The data that support the findings of this study are available on request from the corresponding author. The data are not publicly available due to privacy or ethical restrictions.
